# CD84 Expression Across Disease Stages and Leukemic Subpopulations in Acute Myeloid Leukemia

**DOI:** 10.3390/cancers18183037

**Published:** 2026-09-18

**Authors:** Beatriz Martín-Herreros, Lourdes Cordón, Rebeca Rodríguez-Veiga, Isabel Cano-Ferri, Evelyn Acuña-Cruz, Laura Torres-Miñana, Irene Navarro, Pilar Lloret-Madrid, Amparo Sempere, María Dolores Linares, Sara Torres-Sánchez, Elisa González-Romero, Nela Klein-González, Lorena Pérez-Amill, Leonor Senent, José Luis Poveda-Andrés, Javier De La Rubia, Pau Montesinos, Manuel Guerreiro

**Affiliations:** 1 Hematology Research Group, Instituto de Investigación Sanitaria La Fe (IIS La Fe), 46026 Valencia, Spain; 2 Centro de Investigación Biomédica en Red de Cáncer (CIBERONC), 28029 Madrid, Spain; 3 Hematology Department, Hospital Universitari i Politècnic La Fe, 46026 Valencia, Spain; 4 Fundació de Recerca Clínic Barcelona, Institut d’Investigacions Biomèdiques August Pi I Sunyer (FRCB-IDIBAPS), 08036 Barcelona, Spain; 5 Gyala Therapeutics SL, 08007 Barcelona, Spain; 6 Pharmacy Department, Hospital Universitari i Politècnic La Fe, 46026 Valencia, Spain; 7 Pharmacy Research Group, Instituto de Investigación Sanitaria La Fe (IIS La Fe), 46026 Valencia, Spain; 8 Management Department, Hospital Universitari i Politècnic La Fe, 46026 Valencia, Spain

**Keywords:** acute myeloid leukemia, CD84, CAR T-cell therapy, multiparametric flow cytometry

## Abstract

Background/Objectives: In recent years, increasing evidence has supported CD84 as a promising therapeutic target in acute myeloid leukemia (AML), including its potential application in CAR T-cell therapy. CD84 is consistently reported to be highly expressed on AML cells in both adult and pediatric patients, providing a strong biological rationale for the development of CD84-directed immunotherapies. Methods: We characterized CD84 expression in bone marrow and peripheral blood samples from 61 adult patients with AML at diagnosis, relapse, and refractory disease using multiparametric flow cytometry. Results: Our results demonstrate that CD84 is highly expressed on AML cells throughout the course of the disease. More than 80% of leukemic populations showed CD84 expression above 80%, with median expression levels remaining consistently high at diagnosis (98%), relapse (96.5%), and refractory disease (96%). A novel aspect of our study is the evaluation of CD84 expression across distinct leukemic subpopulations within individual samples; only three of the 94 leukemic populations analyzed showed CD84 expression below 20%. Similarly, CD84 expression remained consistently high across the major AML subtypes analyzed, including AML with NPM1 mutation, AML with myelodysplasia-related gene mutations, and TP53-mutated AML. Comparable median CD84 expression was observed in bone marrow and peripheral blood; however, paired analysis identified significant differences between both compartments. CD84 expression assessed with antibody clone 153-4D9 was significantly lower than that detected with clone CD84.1.21 across AML leukemic populations (median, 75% vs. 98%; *p* < 0.0001). When compared with CD33 and CD123, CD84 showed a similarly broad prevalence of expression across AML leukemic populations. Healthy bone marrow samples were additionally evaluated to characterize the physiological distribution of CD84 expression across normal hematopoietic populations and provide a framework for the assessment of CD84-targeted therapeutic strategies. Conclusions: Together with the available preclinical and clinical evidence, these findings support the continued evaluation of CD84-directed immunotherapies, including CAR T-cell therapy, in AML.

## Simple Summary

Acute myeloid leukemia (AML) remains a challenging disease, and new therapeutic targets are needed to improve patient outcomes. CD84 has recently emerged as a promising target for immunotherapeutic approaches, including CAR T-cell therapy. In this study, we evaluated CD84 expression in samples from adult patients with AML at diagnosis, relapse, and refractory disease. We found that CD84 was consistently expressed across different disease stages, AML subtypes, and leukemic subpopulations. We also compared CD84 with established AML targets and characterized its expression in normal bone marrow populations. These findings provide a comprehensive overview of CD84 expression in AML and further support the continued investigation of CD84-directed immunotherapies.

## 1. Introduction

Acute myeloid leukemia (AML) is a biologically and clinically heterogeneous hematological malignancy arising from the clonal proliferation of myeloid hematopoietic precursor cells harboring diverse genetic and molecular abnormalities [1,2,3]. The disease is characterized by considerable genetic, phenotypic, and clinical variability, which contributes to differences in treatment response and patient outcomes. Despite high initial complete remission (CR) rates following intensive chemotherapy and the potentially curative role of allogeneic hematopoietic stem cell transplantation, relapse continues to be associated with dismal outcomes in AML [4,5]. Moreover, post-relapse survival has remained largely unchanged for decades [6,7], underscoring the urgent need for more effective treatment strategies.

Chimeric antigen receptor T-cell (CAR-T) therapy has emerged as a novel therapeutic approach for B-cell neoplasms. Its remarkable clinical success in relapsed or refractory B-cell acute lymphoblastic leukemia and B-cell lymphomas has demonstrated the therapeutic potential of genetically engineered T-cells and has transformed the treatment landscape of these hematological malignancies [8,9,10], thereby establishing adoptive cellular immunotherapy as a new therapeutic paradigm in hematologic cancers, and encouraged the development of similar approaches in AML [11,12,13,14].

CAR T-cell therapy for AML has been extensively investigated in recent years, with multiple target antigens being evaluated to improve efficacy while minimizing off-tumor toxicity [12,15]. However, CAR-T-based immunotherapy in AML faces challenges that are not typically encountered in B-cell malignancies. This is largely attributable to the biological complexity of the disease, including the heterogeneous expression of target antigens, the presence of an immunosuppressive bone marrow microenvironment, and multiple mechanisms that impair CAR T-cell persistence and functionality, ultimately limiting the durability of antileukemic responses [13].

One of the main challenges for the successful implementation of CAR T-cell therapy in AML is the absence of a truly leukemia-specific target antigen, as most currently investigated targets, including CD33, CD123, CLL-1 and FLT3 [16], among others, are not exclusively expressed on leukemic cells but are also present on normal hematopoietic stem and progenitor cells, and in some cases, mature blood cells, raising concerns regarding on-target/off-tumor toxicity, prolonged myelosuppression and severe hematologic toxicity [17,18]. Furthermore, the marked genetic and phenotypic heterogeneity of AML contributes to variable antigen expression and increases the risk of tumor escape through antigen loss or downregulation [19]. Additional barriers include the immunosuppressive bone marrow microenvironment, impaired CAR T-cell persistence, and limited activity against leukemia stem cells, which are believed to contribute to disease relapse and therapeutic resistance [20,21]. As the field continues to evolve, significant efforts have focused on the identification of novel target antigens capable of overcoming these limitations while preserving an acceptable safety profile. Among these, CD84 has emerged as a promising therapeutic target. CD84 is a transmembrane receptor belonging to the signaling lymphocyte activation molecule (SLAM) family that is physiologically expressed on hematopoietic cells but is overexpressed on AML cells at diagnosis and relapse. As a homophilic adhesion molecule, CD84 mediates cell-to-cell interactions within hematopoietic and immune compartments and participates in the regulation of multiple immune processes [22]. Accordingly, several preclinical studies have demonstrated the antileukemic activity and therapeutic potential of CD84-directed CAR T-cells in AML [22,23,24,25,26]. Importantly, CD84 has been reported to be broadly expressed across different AML subtypes, supporting its potential as a target for immunotherapeutic interventions. Furthermore, the persistence of CD84 expression at disease recurrence highlights its potential relevance not only for the treatment of newly diagnosed AML but also for relapsed or refractory disease [23]. Collectively, these findings provide a strong rationale for the further investigation of CD84-targeted immunotherapeutic strategies in AML.

The aim of this study was to comprehensively characterize CD84 expression throughout the AML disease course, including newly diagnosed, relapsed, and refractory disease, using multiparametric flow cytometry. Beyond evaluating overall CD84 expression, we assessed its distribution across distinct immunophenotypically defined leukemic populations within individual AML samples, as well as across different genetic AML subtypes and normal bone marrow compartments. This approach was intended to investigate potential expression heterogeneity across leukemic subsets and to provide a more detailed assessment of its potential applicability as a target for CD84-directed CAR T-cell therapy.

## 2. Material and Methods

### 2.1. Patients

A total of 61 adult patients diagnosed with AML according to the World Health Organization (WHO) classification [2] were included in this study at Hospital Universitario y Politécnico La Fe (Valencia, Spain) between April and November 2024. All cases underwent morphological, immunophenotypic, molecular, and cytogenetic evaluation. Bone marrow and peripheral blood samples were collected as part of the routine diagnostic workup. In addition, to evaluate the potential on-target/off-tumor effects of a CD84-targeted therapy, control bone marrow samples were obtained from five healthy bone marrow donors. Written informed consent was obtained from all participants in accordance with the Declaration of Helsinki and applicable regulations governing biomedical research, bioethics, and data protection.

### 2.2. Multiparametric Flow Cytometry

Multiparametric flow cytometry (MFC) was performed on peripheral blood and/or bone marrow samples collected in EDTA tubes and processed within 36 h from collection. Samples were stained and incubated for 30 min at room temperature in the dark with a mixture of fluorochrome-conjugated monoclonal antibodies including CD84-PE clone CD84.1.21 (BioLegend, San Diego, CA, USA) and clone 153-4D9 (Novus Biologicals, Centennial, CO, USA) or the corresponding isotypic control. The fluorochrome-conjugated monoclonal antibodies used were: CD15 FITC, CD19 FITC, CD64 FITC, CD34 PerCP-Cy5.5, CD13 PE-Cy7, CD33 PE-Cy7, CD117 PE-Cy7, CD11b APC, CD123 APC, CD300e APC, CD10 APC-H7, CD14 APC-H7, CD38 APC-H7, HLA-DR V450, CD45 V500-C (Supplementary Information). The volumes of monoclonal antibodies used for staining were determined according to previously established protocols [23]. After red blood cell lysis with FACS Lysing solution (Becton Dickinson, San Jose, CA, USA) and two washing steps, cell pellets were resuspended in phosphate-buffered saline (PBS) and acquired on a FACSCanto II™ cytometer (Becton Dickinson, San Jose, CA, USA). Instrument setup, fluorescence compensation, and daily quality control procedures were performed according to the manufacturer’s recommendations to ensure consistency and reproducibility of fluorescence measurements throughout the study period. Flow cytometer performance was assessed using Cytometer Setup & Tracking (CS&T) beads (BD Biosciences, San Jose, CA, USA). Spectral overlap compensation settings were established at the beginning of the study and subsequently monitored throughout the study using the assay tube settings setup option of the flow cytometer. Whenever feasible, a minimum of 1 × 10^6^ events was acquired. Data were analyzed using Infinicyt 2.0 software (Cytognos, Salamanca, Spain).

Diagnostic immunophenotyping had been previously established according to EuroFlow standardized operating procedures using the Acute Leukemia Orientation Tube (ALOT) and the AML/ myelodysplastic syndromes (MDS) panel [27,28]. These combinations of fluorochrome-conjugated monoclonal antibodies enabled the identification, phenotypic characterization, and maturation-stage assessment of the distinct leukemic populations present in each sample. CD84 expression was subsequently assessed within the leukemic blast compartment previously defined according to its diagnostic immunophenotypic profile. Samples were analyzed individually and manually, and no batch analysis was performed. The cutoff for CD84 positivity was established individually for each sample using the corresponding isotype control processed in parallel from the same sample as the reference for background fluorescence. Representative examples of the gating strategy used to identify leukemic populations and normal hematopoietic bone marrow populations prior to CD84 assessment are provided in Supplementary Figures S1 and S2, respectively.

### 2.3. Statistical Analysis

The descriptive analysis was performed using percentages for categorical variables and medians (range) for continuous variables. Statistical analysis was carried out using GraphPad Prism version 9 software with tests selected according to the nature and distribution of the variables. Prior to analysis, data distribution was assessed using the Shapiro–Wilk normality test. As the evaluated variables did not consistently follow a normal distribution, non-parametric tests were used throughout the study. Comparisons among multiple groups were performed using the Kruskal–Wallis test. Post hoc multiple comparison analyses were planned when a significant overall difference was identified. Paired comparisons addressing predefined biological questions were analyzed using the Wilcoxon signed-rank test. Statistical significance was defined as *p* < 0.05, and results were interpreted according to this predefined threshold. The corresponding statistical outputs were generated directly using the software package and reviewed before data interpretation. Analyses were performed at patient, sample, or leukemic population level as indicated. In analyses that included different abnormal populations, multiple observations could originate from the same patient. These analyses were intended to characterize CD84 expression across phenotypically defined leukemic subsets and to assess patient heterogeneity. Therefore, observations should not be interpreted as fully independent patient-level measurements.

## 3. Results

### 3.1. Patient Characteristics

We evaluated CD84 expression in 91 samples from 61 adult patients with a median age of 68 years (range, 32–82); 34 were males (56%). We enrolled 37 patients at diagnosis. Paired bone marrow and peripheral blood samples were available for 24 patients, while only one sample type was available for the remaining 13 patients, resulting in a total of 61 diagnostic samples (67%). We also included 11 patients at relapse, one of whom provided both sample types (*n* = 12 samples, 13%), and 18 patients with refractory disease, each contributing either a bone marrow or a peripheral blood sample (*n* = 18 samples, 20%). Forty-one samples were analyzed using both CD84 antibody clones (CD84.1.21, Biolegend and 153-4D9, Novus) (Supplementary Information). Moreover, longitudinal changes in CD84 expression were evaluated in five patients with samples available from different disease stages, including four patients with paired diagnosis and refractory samples and one patient with paired relapse and refractory samples. Healthy donors included four males and one female, with a median age of 50 years (range, 37–56 years).

### 3.2. CD84 Expression in AML Across Disease Stages

**Figure 1:**
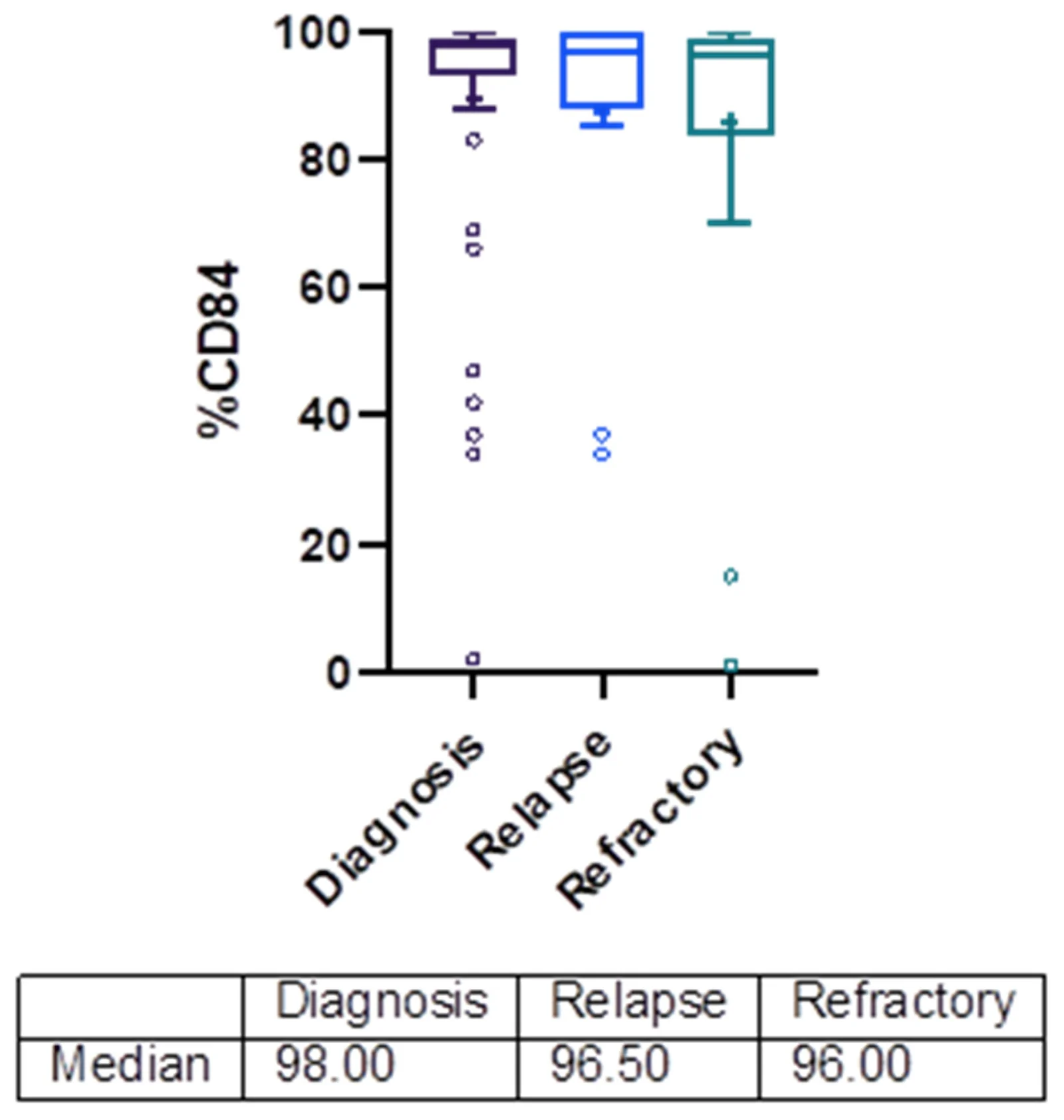
CD84 expression (clone CD84.1.21) measured by multiparametric flow cytometry in bone marrow and peripheral blood samples from 61 patients with AML at diagnosis, relapse, and refractory disease. A total of 66 samples were included in this analysis; when both peripheral blood and bone marrow samples were available from the same patient at the same disease stage, bone marrow samples were selected for analysis. Five of the patients had samples available from different disease stages, including four patients with paired diagnosis and refractory samples and one patient with paired relapse and refractory samples. Individual data points represent CD84 expression in each leukemic population identified (diagnosis, *n* = 55 leukemic populations from 37 patients; relapse, *n* = 14 leukemic populations from 11 patients; refractory disease, *n* = 25 leukemic populations from 18 patients). Data are presented as Tukey box-and-whisker plots showing the median, interquartile range, whiskers, individual outliers, and mean (+). Statistical comparisons were performed using the Kruskal–Wallis test. No statistically significant differences were observed (*p* = 0.31).

CD84 expression (clone CD84.1.21) was assessed in individual leukemic populations identified by MFC from samples obtained at different stages of the disease. When multiple phenotypically distinct neoplastic cell populations were present in a single patient sample, each population was analyzed independently rather than using a single bulk expression value. MFC analysis demonstrated a median CD84 expression of 98% (range, 2–100%) at diagnosis, 96.5% (range, 34–100%) at relapse, and 96% (range, 1–100%) in refractory disease (Figure 1). High CD84 expression (>80% positive leukemic cells) was observed in more than 80% of neoplastic cell populations across the cohort (Table 1).

### 3.3. CD84 Expression in Distinct Leukemic Subpopulations and AML Subtypes

**Figure 2:**
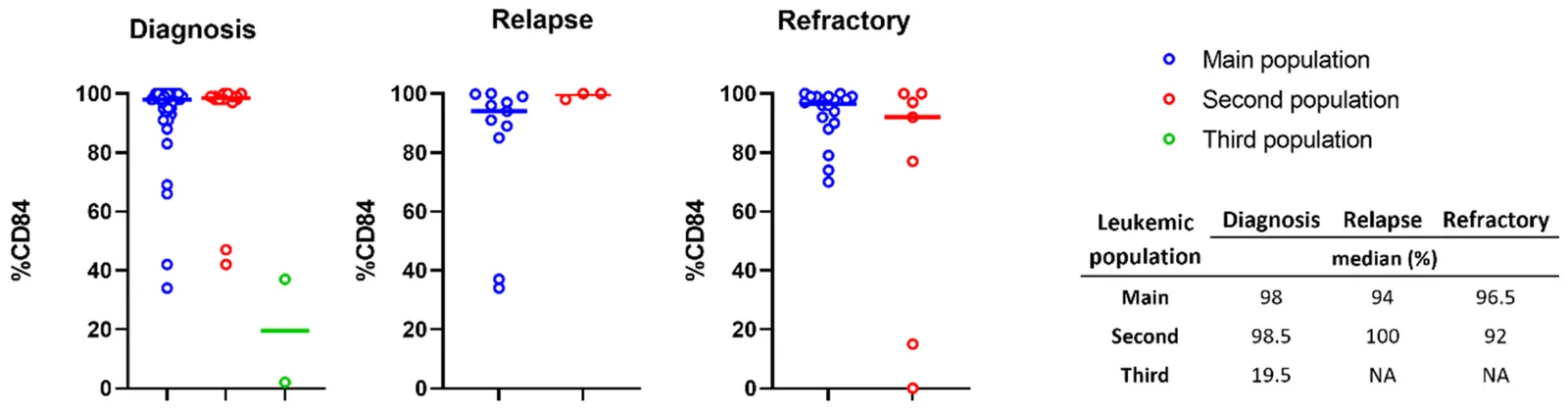
CD84 expression (clone CD84.1.21) measured by multiparametric flow cytometry (MFC) in distinct leukemic populations identified within bone marrow and peripheral blood samples from 61 patients with AML at diagnosis, relapse, and refractory disease. Leukemic populations were classified as the main, the second, or the third according to the MFC gating strategy. A total of 66 samples were included in this analysis, when both peripheral blood and bone marrow samples were available from the same patient at the same disease stage, bone marrow samples were selected for analysis. Five of the patients have samples available from different disease stages, including four patients with paired diagnosis and refractory samples and one patient with paired relapse and refractory samples. Individual data points represent CD84 expression in each leukemic population identified (diagnosis: main, *n* = 37; second, *n* = 16; third, *n* = 2, *n* total = 55 leukemic populations from 37 patients; relapse: main, *n* = 11; second, *n* = 3, *n* total = 14 leukemic populations from 11 patients; refractory disease: main, *n* = 18; second, *n* = 7, *n* total = 25 leukemic populations from 18 patients). The dataset used for this analysis is the same as that described in Figure 1 and Table 1.

**Figure 3:**
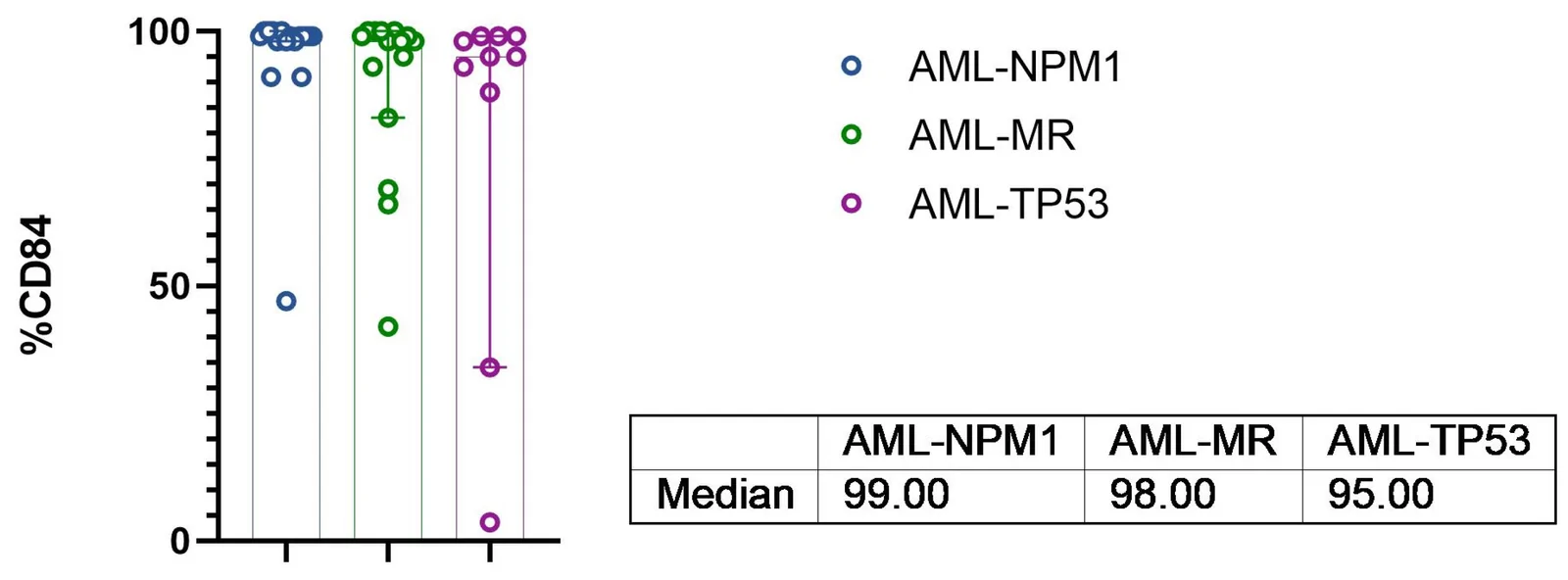
Comparison of CD84 expression (clone CD84.1.21) in leukemic populations at diagnosis according to AML subtype based on the WHO classification [2] in 28 patients. Only the most prevalent AML subtypes in our cohort were included in this analysis due to the limited number of cases in the remaining categories. AML subtypes included AML with *NPM1* mutation (AML-*NPM1*, 16 leukemic populations from 11 patients), AML with myelodysplasia-related gene mutations excluding *TP53* mutations (AML-MR, 15 leukemic populations from 11 patients), and AML with mutated *TP53* (AML-*TP53*, 10 leukemic populations from 6 patients). Individual data points represent CD84 expression in each leukemic population identified.

To investigate the impact of leukemic cell heterogeneity, CD84 expression (clone CD84.1.21) was analyzed in distinct AML cell subpopulations identified within each sample. At diagnosis, a single leukemic population was identified in 21 (57%) patients, while 16 (43%) patients had more than one leukemic population, including two patients with three distinct neoplastic cell populations (Figure 2, Supplementary Table S2). Median CD84 expression was 98%, 98%, and 19.5% in the main, the second, and the third leukemic population, respectively. At diagnosis, only one blast cell population showed CD84 expression < 20% (Table 1), a threshold that has historically been used in MFC to define positive antigen expression for several immunophenotypic markers. At relapse, three patients had two neoplastic cell populations (Figure 2, Supplementary Table S2), with median CD84 expression of 94% and 100% in the main and the second leukemic populations, respectively. In refractory disease, seven patients had two blast populations (Figure 2, Supplementary Table S2), with a median CD84 expression of 96.5% in the main and 92% in the second leukemic population. Only two leukemic populations identified in refractory disease showed CD84 expression < 20% (Table 1). Because the primary objective of this study was to assess the prevalence of CD84 expression and identify targetable leukemic populations, the main analyses are presented as the percentage of CD84-positive cells. Normalized median fluorescence intensity (nMedFI) data are provided in Supplementary Table S2. Finally, we compared CD84 expression at diagnosis across different AML subtypes according to WHO classification. A total of 16 leukemic populations from 11 patients with AML with *NPM1* mutation showed a median CD84 expression of 99% (range, 47–100). In AML with myelodysplasia-related gene mutations (excluding *TP53* mutations), 15 leukemic populations from 11 patients displayed a median expression of 98% (range, 42–100). Likewise, 10 leukemic populations from 6 patients with AML with mutated *TP53* showed a median CD84 expression of 95% (range, 3.6–99) (Figure 3).

### 3.4. Longitudinal Assessment of CD84 Expression

Longitudinal changes in CD84 expression (clone CD84.1.21) were evaluated in the five patients with samples available at different disease stages (Table 2). Three patients (patients 8, 33, and 42) had paired samples from diagnosis and refractory disease and showed comparable CD84 expression levels at both time points. In patient 32, CD84 expression remained stable in one leukemic population between relapse and refractory disease, whereas the second leukemic population showed reduced expression at the refractory stage, moving from 37% to absence of expression. In patient 58, CD84 expression decreased from diagnosis to refractory disease in both the main and secondary leukemic populations (42% vs. 15% and 100% vs. 90%, respectively).

### 3.5. Comparison of CD84 Expression Across Sample Types and Antibody Clones

**Figure 4:**
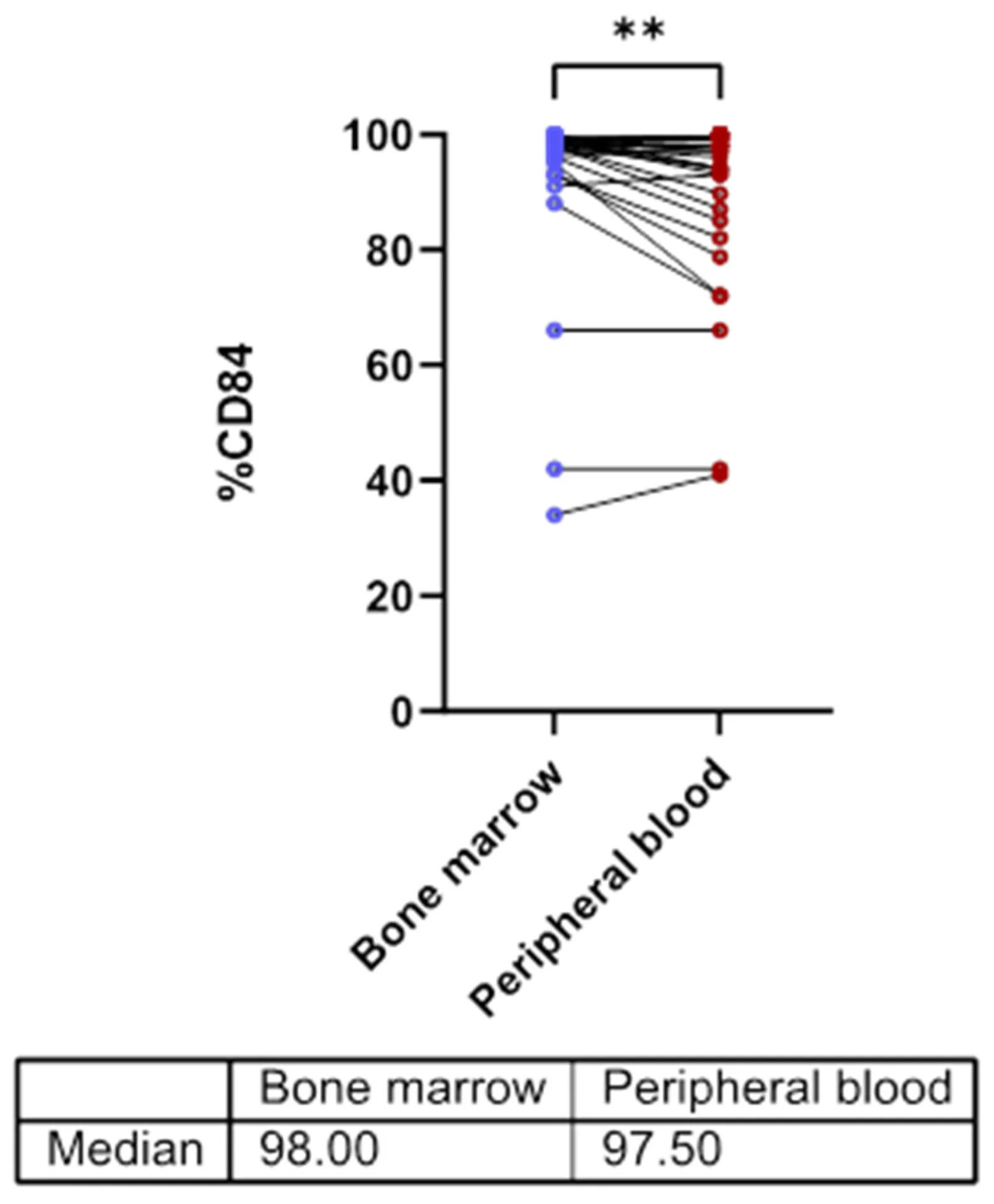
Comparison of CD84 expression (clone CD84.1.21) at diagnosis between paired bone marrow and peripheral blood samples from 24 AML patients for whom both samples were available at diagnosis. Individual data points represent CD84 expression in each paired leukemic population (*n* = 36), as 12 patients presented two distinct leukemic populations in both samples. Statistical comparisons were performed using the paired Wilcoxon signed-rank test. ** *p* = 0.0055.

To assess the influence of sample source, CD84 expression (clone CD84.1.21) was compared in paired bone marrow and peripheral blood samples obtained from patients with AML at diagnosis (Figure 4). Median CD84 expression was similar in bone marrow (98%; range, 34–100) and peripheral blood (97.5%; range, 41–100). However, despite these comparable median values, paired statistical analysis demonstrated a significant difference between the two sample types (*p* = 0.0055).

**Figure 5:**
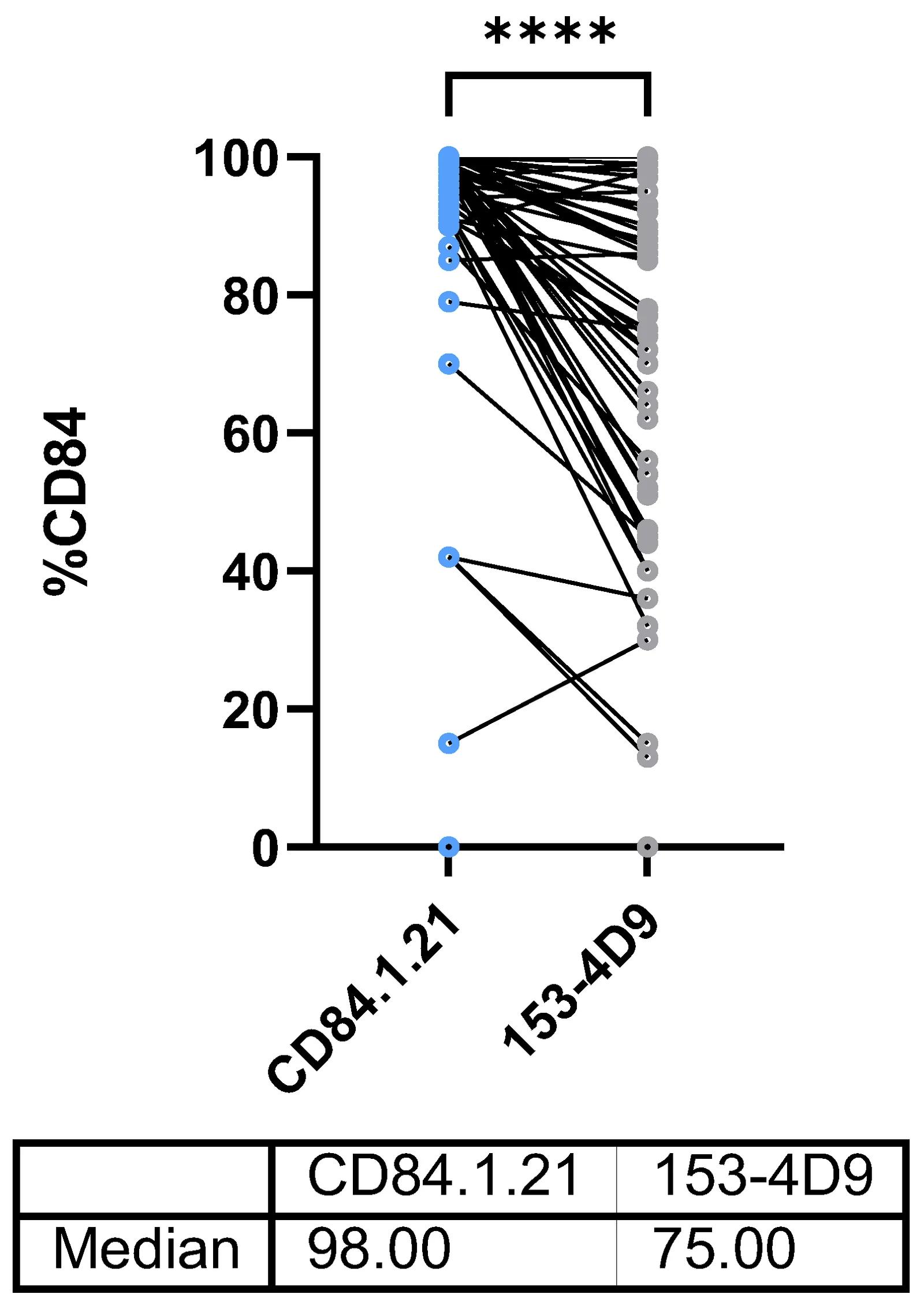
CD84 expression assessed with antibody clones CD84.1.21 and 153-4D9 in paired leukemic populations from bone marrow and peripheral blood samples collected at diagnosis, relapse, and refractory disease stages from 32 AML patients. Statistical comparisons were performed using the paired Wilcoxon signed-rank test. **** *p* < 0.0001. Individual data points represent leukemic populations analyzed with both antibody clones (*n* = 54), including 33 populations at diagnosis, 7 at relapse, and 14 at refractory disease.

CD84 expression was additionally evaluated using an alternative antibody clone (153-4D9). Comparison of both clones in the same leukemic populations across different disease stages (diagnosis, relapse, and refractory disease) and sample types (bone marrow and peripheral blood) revealed significantly lower expression with clone 153-4D9 than with clone CD84.1.21 (median, 75% vs. 98%, respectively; *p* < 0.0001, Figure 5).

### 3.6. Comparison of CD84 Expression with Established AML Therapeutic Targets

**Figure 6:**
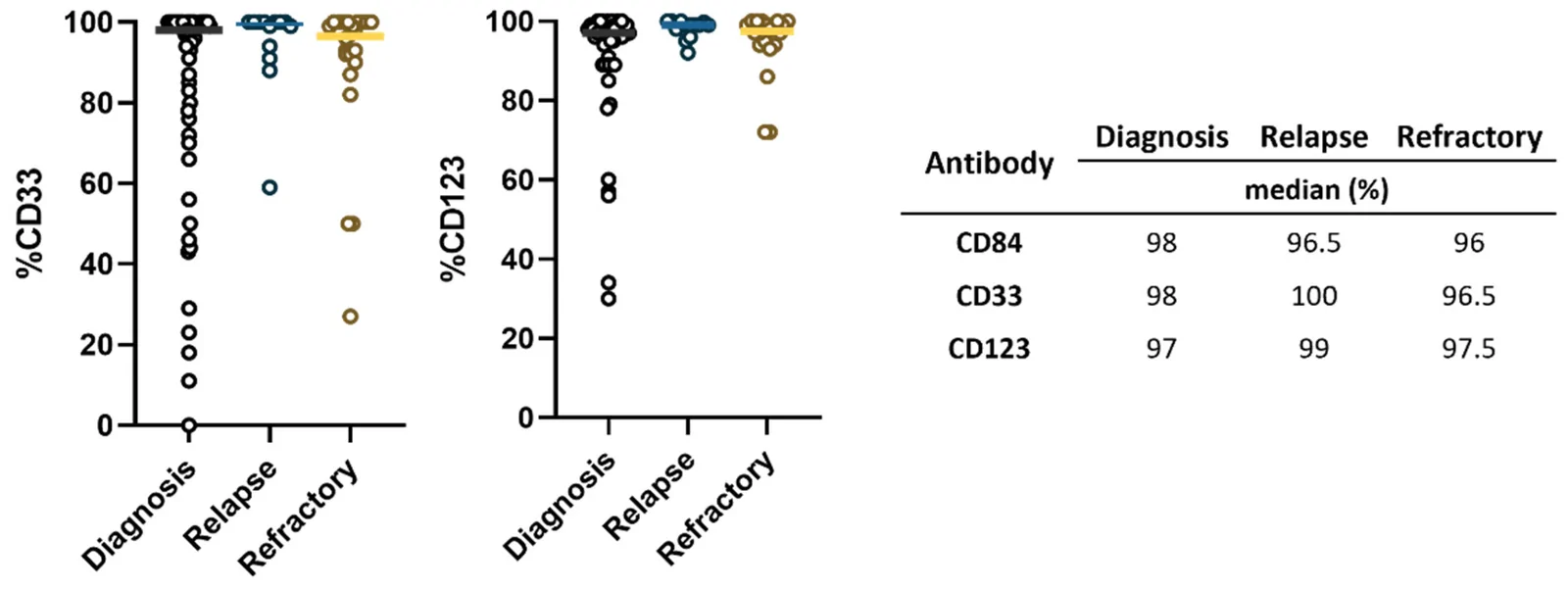
Expression of CD33 and CD123 measured by multiparametric flow cytometry in bone marrow and peripheral blood samples from 61 patients with AML at diagnosis, relapse, and refractory disease. Individual data points represent the expression of CD33 and CD123 in each leukemic population identified (diagnosis: *n* = 55 and 54; relapse: *n* = 13 for both markers; refractory disease: *n* = 24 and 22, respectively). Colored solid lines indicate the median. The dataset used for this analysis is the same as that described in Figure 1; differences in *n* values are due to non-evaluable CD33 and/or CD123 expression data in some leukemic populations.

To place CD84 expression in the context of established AML targets, we compared its expression with that of CD33 and CD123 across the same disease stages (Figure 6). Median CD33 expression was 98% (range, 0–100) at diagnosis, 100% (range, 59–100) at relapse, and 96.5% (range, 27–100) in refractory disease. Corresponding median CD123 expression was 97% (range, 30–100), 99% (range, 92–100), and 97.5% (range, 72–100), respectively. Overall, CD33 and CD123 expression levels across all disease stages were comparable to CD84 expression levels, as described in the previous section.

### 3.7. CD84 Expression in Normal Hematopoietic Populations

**Figure 7:**
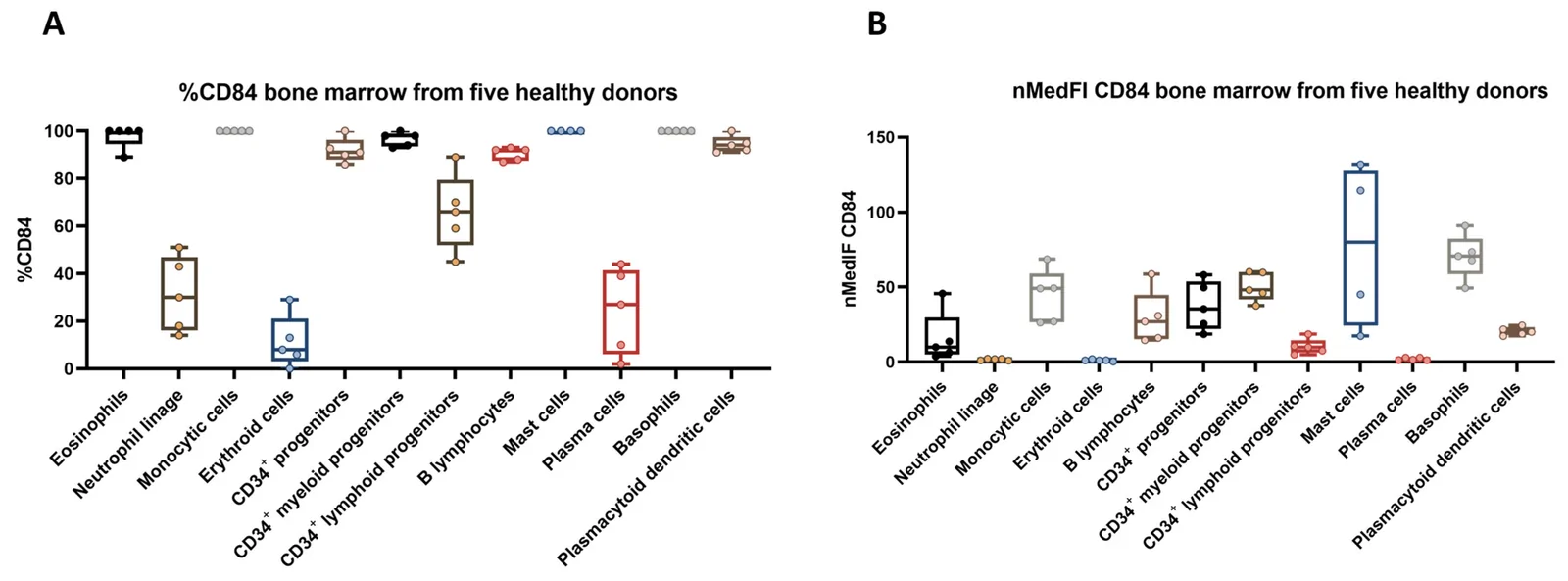
CD84 expression (clone CD84.1.21) in different hematopoietic populations from the bone marrow of five healthy donors. Data are presented as the percentage of CD84-positive cells (**A**) and the normalized median fluorescence intensity (nMedFI) (**B**). CD84 nMedFI was calculated as the ratio of the median fluorescence intensity between the CD84-specific antibody and its corresponding isotypic control.

To assess potential on-target/off-tumor effects of a CD84-targeted therapy, CD84 expression (clone CD84.1.21) was evaluated in different hematopoietic populations from the bone marrow of five healthy donors using both the percentage of positive cells and (nMedFI) as complementary measures (Figure 7). CD84 expression showed a distinct distribution pattern among normal hematopoietic cell populations analyzed. The highest percentages of CD84-positive cells were detected in eosinophils, monocytes, B lymphocytes, mast cells, basophils, plasmacytoid dendritic cells, and CD34+ myeloid progenitor cells. In contrast, neutrophils, erythroid cells, and plasma cells showed more variable and generally lower percentages of expression. Assessment of CD84 expression intensity yielded similar results, with higher nMedFI values observed in mast cells, basophils, monocytes, CD34+ myeloid progenitor cells, and B lymphocytes compared with neutrophils, erythroid cells, and plasma cells. Despite some degree of inter-donor variability being observed, particularly among mast cells and progenitor populations, the overall expression profile remained consistent across all healthy donors analyzed.

## 4. Discussion

AML remains a highly aggressive hematologic malignancy associated with poor clinical outcomes despite recent therapeutic advances [1,2,3,4,5,6,7]. While CAR T-cell therapy has dramatically improved the management of several B-cell malignancies, its application in AML has been considerably more challenging due to the absence of an ideal target antigen. The heterogeneous biology of AML and the significant overlap between leukemic and normal hematopoietic cells increase the risk of on-target/off-tumor toxicity, which has limited the clinical development of CAR-T strategies in this disease [17,19]. Therefore, there is a strong need to identify novel antigens that are consistently expressed across AML cells while maintaining an acceptable safety profile in normal hematopoietic tissues.

In recent years, increasing evidence has supported CD84 as a promising therapeutic target in AML, including its potential application in CAR T-cell therapy [22,23,24,25,26,29]. CD84 is consistently reported to be highly expressed on AML cells in both adult and pediatric patients, providing a strong biological rationale for the development of CD84-directed immunotherapies. In addition, the growing interest in CD84 arises from the persistent need to identify novel AML-associated antigens that can overcome some of the limitations associated with currently available targets. Therefore, a detailed characterization of CD84 expression across the biological diversity of AML is essential to further define its therapeutic potential and applicability in different stages of the disease.

In the present study, we characterized CD84 expression in bone marrow and peripheral blood samples from 61 adult patients with AML at diagnosis, relapse, and refractory disease using MFC. This widely available technique provides a rapid, reproducible, and cost-effective approach for evaluating CD84 expression and could be readily incorporated into the screening of patients considered for CD84-directed clinical trials.

Our results demonstrate that CD84 is highly expressed on AML cells throughout the course of the disease. More than 80% of leukemic populations showed CD84 expression above 80%, with median expression levels remaining consistently high at diagnosis (98%), relapse (96.5%), and refractory disease (96%). These findings are consistent with previous reports describing high CD84 expression in AML. Pigazzi et al. demonstrated high CD84 expression in 99% of patients from an independent validation cohort and further showed that CD84 expression was maintained across diagnosis, and relapse. Notably, expression was also retained in all evaluable measurable residual disease (MRD) samples and in the vast majority of other myeloid malignancies [24,25]. Similarly, Zhu et al. reported CD84 positivity (>70%) in more than half of AML samples [26]. The higher proportion of CD84-positive leukemic populations observed in our study compared with the latter study is likely explained by our MFC approach, which evaluated CD84 expression in individual leukemic subpopulations rather than across the entire neoplastic cell compartment of each sample.

A novel aspect of our study is the evaluation of CD84 expression across distinct leukemic subpopulations within individual samples. While previous studies have mainly focused on overall CD84 expression at the bulk leukemic population level, we sought to determine whether antigen expression was maintained across all phenotypically distinct leukemic compartments identified within each case. This issue is particularly relevant in the context of CAR T-cell therapy, where antigen escape has emerged as one of the major mechanisms of treatment failure [13,18,19,20]. Under selective therapeutic pressure, minor antigen-negative or antigen-low subclones may survive treatment and subsequently expand, ultimately contributing to disease persistence and relapse. Therefore, the identification of target antigens that are homogeneously expressed not only in the main leukemic population but also in minor subpopulations is a critical prerequisite for achieving durable therapeutic response. Because the presence of CD84-negative clones may impair therapeutic responses, the expansion or clonal evolution of these populations could hinder the achievement of the complete remission required before consolidation with allogeneic hematopoietic stem cell transplantation (allo-HSCT).

To our knowledge, this has not been systematically investigated previously. Although only three of the 94 leukemic populations analyzed showed CD84 expression below 20%, these findings highlight the importance of assessing antigen expression across all leukemic subsets, including minor populations, as immunophenotypic heterogeneity may influence the suitability of a therapeutic target. A threshold of ≥20% positivity was selected as a study-specific threshold because it is currently used as an inclusion criterion in the ongoing clinical trial with CART-84 (EU trial No.: 2024-519966-31-00). Although no validated biological cutoff for CD84 expression has been established in AML, percentage-based cutoffs around 20% have historically been applied in certain immunophenotypic classification systems to define antigen expression by MFC, although marker-specific interpretation remains context dependent.

While CD84 expression was broadly maintained across diagnosis, relapse, and refractory disease at the cohort level, the limited number of patients with longitudinal samples precludes definitive conclusions regarding expression stability within individual patients. Furthermore, the reductions observed in some leukemic populations may reflect biological processes such as clonal evolution, antigen downregulation, or the expansion of pre-existing CD84-low populations that were below the limit of detection at earlier disease stages.

Beyond the heterogeneity studied within individual leukemic samples, our analysis across different genetic AML categories is especially relevant given the recognized biological and immunophenotypic heterogeneity of the disease. As shown in AML with *NPM1* mutation, distinct leukemic populations with different maturation stages may coexist within the same patient [30,31], emphasizing the importance of evaluating potential CAR-T targets across diverse AML subtypes and cellular compartments. CD84 expression remained consistently high across the major AML subtypes analyzed, including AML with *NPM1* mutation, AML with myelodysplasia-related gene mutations, and *TP53*-mutated AML. These observations are consistent with previous reports demonstrating preserved CD84 expression across AML genetic subgroups [23,25]. Pigazzi et al. did not identify a clear association between CD84 expression and specific molecular AML subtypes, although they noted a trend toward higher expression in *KMT2A* and *NUP98* rearranged cases. Similarly, CD84 expression remained preserved across the different genetic categories evaluated in our cohort, further supporting the notion that its expression is largely maintained regardless of the underlying molecular background. Although a limited number of *KMT2A*-rearranged and *NUP98*-rearranged cases were included in our series, precluding definitive conclusions regarding these specific subgroups, our findings further support the notion that CD84 expression is largely maintained regardless of the underlying molecular background. However, the results observed across AML genetic subgroups should be interpreted with caution due to the relatively small and unequal sample sizes among the different categories.

Together with the consistent expression observed across distinct leukemic subpopulations within individual samples, these results suggest that CD84 expression is preserved across multiple layers of AML heterogeneity, including both genetic diversity and phenotypic complexity. This broad expression across both different AML genetic backgrounds and leukemic cellular compartments is particularly relevant in the context of CAR T-cell therapy, as it may facilitate more comprehensive target coverage throughout the leukemic architecture and potentially reduce the likelihood of treatment failure driven by pre-existing antigen-negative subclones. Collectively, these findings further support the applicability of CD84-directed therapies across a genetically diverse population of AML patients and reinforce the rationale for the continued development of CD84-targeted immunotherapeutic strategies.

An additional observation from this study was the lower CD84 expression detected using the antibody clone 153-4D9 compared with CD84.1.21. Despite these quantitative differences, CD84 remained broadly expressed across AML samples regardless of the clone used. However, differences in clone performance may be relevant when applying a predefined threshold for patient eligibility for CD84-directed CAR T-cell therapy, particularly in patients with expression close to the cutoff. These findings emphasize the importance of antibody clone selection and methodological standardization when assessing CD84 expression by MFC. Similar to previous studies evaluating the expression profile of established AML targets such as CD33 and CD123 [16,32,33], broad and consistent antigen expression represents one of the main prerequisites for the successful development of immunotherapeutic approaches. To further characterize CD84 expression in AML, we compared its prevalence of expression with that of the established AML target antigens CD33 and CD123. CD84 showed a similar prevalence of expression across leukemic populations at all disease stages. While antigen density and functional activity were not assessed in the present study, our findings demonstrate broad CD84 expression across AML populations and complement previous studies evaluating the therapeutic potential of CD84-directed approaches.

Finally, analysis of healthy bone marrow samples confirmed the previously described physiological expression pattern of CD84, with highest expression on monocytes, B lymphocytes, CD34+ myeloid progenitors, mast cells, and basophils. These findings are consistent with previous studies [22,23] and provide additional information regarding the normal hematopoietic distribution of CD84 that should be considered during the clinical development of CD84-directed immunotherapies. Although CD84 expression was observed in normal hematopoietic populations, including CD34+ myeloid progenitors, these findings do not exclude the possibility of on-target/off-tumor effects following CD84-directed therapy. Therefore, careful assessment of hematologic toxicity will be required in the ongoing clinical trials (EU Trial No. 2024-519966-31-00). Notably, the study protocol includes allo-HSCT after CAR T-cell infusion, acknowledging the potential risk of myelotoxicity and providing a rescue strategy should prolonged hematopoietic toxicity occur.

Our study has several limitations, including its retrospective single-center design and the relatively limited number of relapse and refractory samples. Consequently, the results should be interpreted with appropriate caution and would benefit from validation in larger, independent cohorts. In addition, the reduced representation of relapsed and refractory cases limits the ability to draw definitive conclusions regarding CD84 expression in these specific clinical settings. Moreover, the analyses performed at the leukemic population level, which allow multiple observations of the same patient, could be considered a limitation. Consequently, these observations are not entirely independent, and the results should be interpreted with an understanding of the descriptive and exploratory nature of the study. It should also be acknowledged that the number of healthy bone marrow samples included in the study was relatively small, which may limit the assessment of variability in the physiological expression pattern of CD84 among healthy donors. Nevertheless, it represents, to our knowledge, the most comprehensive MFC characterization of CD84 expression across leukemic subpopulations. Furthermore, the inclusion of normal bone marrow samples enabled a detailed assessment of CD84 expression in non-malignant hematopoietic compartments, providing additional information regarding the potential safety profile of CD84-targeted therapeutic approaches. The broad spectrum of samples analyzed, together with the evaluation of distinct leukemic populations within individual cases, strengthens the translational relevance of our findings and provides additional support for the ongoing clinical development of CD84-directed immunotherapies in AML. In this context, the results of ongoing clinical studies will help determine the clinical impact of CD84 targeting and clarify its potential role within the evolving immunotherapeutic landscape of AML.

## 5. Conclusions

Overall, our findings demonstrate that CD84 is broadly and consistently expressed in AML, irrespective of disease stage, genetics, or leukemic subpopulation. The preservation of CD84 expression across the different leukemic compartments analyzed is particularly relevant in the context of CAR T-cell therapy, as it suggests that CD84 may be capable of providing broad target coverage despite the phenotypic heterogeneity of AML. Together with its expression profile relative to established AML targets and previously reported preclinical evidence supporting CD84-directed immunotherapies, these observations further support CD84 as a promising immunotherapeutic target in AML and provide additional rationale for the continued development and clinical evaluation of CD84-directed therapies, including CAR T-cell strategies.

## Figures and Tables

**Table 1:** Expression levels of CD84 (clone CD84.1.21) in the different leukemic populations identified in samples from AML newly diagnosed, relapsed or refractory patients. The dataset used for this analysis is the same as that described in Figure 1.

%CD84	Diagnosis (*n* = 55)	%	Relapse (*n* = 14)	%	Refractory (*n* = 25)	%	Total (*n* = 94)	%
>80	47	85.5	12	85.7	19	76.0	78	83.0
21–79	7	12.7	2	14.3	4	16.0	13	13.8
<20	1	1.8	0	0	2	8.0	3	3.2

**Table 2:** Longitudinal comparison of CD84 (clone CD84.1.21) expression in the five patients with samples available at different disease stages. LINK Excel.Sheet.12.

ID	Sample	Population	%CD84		
			Diagnosis	Relapse	Refractory
32	BM	1	NA	98	97
	BM	2	NA	37	0
8	PB	1	69	NA	70
33	BM	1	95	NA	99
42	BM	1	97	NA	92
	BM	2	99	NA	96
58	PB	1	42	NA	15
	PB	2	100	NA	90

BM, bone marrow. PB, peripheral blood. NA, not applicable.

## Data Availability

The original contributions presented in this study are included in the article. Further inquiries can be directed to the corresponding author.

## Supplementary Materials

The following supporting information can be downloaded at: https://www.mdpi.com/article/10.3390/cancers18183037/s1, Table S1: Monoclonal antibody information; Table S2: Patient characteristics and CD84 (clone CD84.1.21) expression in the leukemic populations. Rows highlighted in grey indicate the same patient; Figure S1: A representative flow cytometry image of the sequential gating strategy in a patient with three distinct leukemic populations; Figure S2: Representative flow cytometry plots showing the identification of the main normal hematopoietic populations in healthy bone marrow samples.

## References

[1] Papaemmanuil E., Gerstung M., Bullinger L., Gaidzik V.I., Paschka P., Roberts N.D., Potter N.E., Heuser M., Thol F., Bolli N. (2016). Genomic Classification and Prognosis in Acute Myeloid Leukemia. N. Engl. J. Med..

[2] Khoury J.D., Solary E., Abla O., Akkari Y., Alaggio R., Apperley J.F., Bejar R., Berti E., Busque L., Chan J.K.C. (2022). The 5th Edition of the World Health Organization Classification of Haematolymphoid Tumours: Myeloid and Histiocytic/Dendritic Neoplasms. Leukemia.

[3] Arber D.A., Orazi A., Hasserjian R.P., Borowitz M.J., Calvo K.R., Kvasnicka H.-M., Wang S.A., Bagg A., Barbui T., Branford S. (2022). International Consensus Classification of Myeloid Neoplasms and Acute Leukemias: Integrating Morphologic, Clinical, and Genomic Data. Blood.

[4] Kreidieh F., Abou Dalle I., Moukalled N., El-Cheikh J., Brissot E., Mohty M., Bazarbachi A. (2022). Relapse after Allogeneic Hematopoietic Stem Cell Transplantation in Acute Myeloid Leukemia: An Overview of Prevention and Treatment. Int. J. Hematol..

[5] Megías-Vericat J.E., Martínez-Cuadrón D., Sanz M.Á., Montesinos P. (2018). Salvage Regimens Using Conventional Chemotherapy Agents for Relapsed/Refractory Adult AML Patients: A Systematic Literature Review. Ann. Hematol..

[6] Ganzel C., Sun Z., Cripe L.D., Fernandez H.F., Douer D., Rowe J.M., Paietta E.M., Ketterling R., O’Connell M.J., Wiernik P.H. (2018). Very Poor Long-term Survival in Past and More Recent Studies for Relapsed AML Patients: The ECOG-ACRIN Experience. Am. J. Hematol..

[7] Shimony S., Stahl M., Stone R.M. (2025). Acute Myeloid Leukemia: 2025 Update on Diagnosis, Risk-Stratification, and Management. Am. J. Hematol..

[8] Cappell K.M., Kochenderfer J.N. (2023). Long-Term Outcomes Following CAR T Cell Therapy: What We Know so Far. Nat. Rev. Clin. Oncol..

[9] Ortiz-Maldonado V., Alonso-Saladrigues A., Español-Rego M., Martínez-Cibrián N., Faura A., Magnano L., Català A., Benítez-Ribas D., Giné E., Díaz-Beyá M. (2022). Results of ARI -0001 CART19 Cell Therapy in Patients with Relapsed/Refractory CD19 -positive Acute Lymphoblastic Leukemia with Isolated Extramedullary Disease. Am. J. Hematol..

[10] Zugasti I., Espinosa-Aroca L., Fidyt K., Mulens-Arias V., Diaz-Beya M., Juan M., Urbano-Ispizua Á., Esteve J., Velasco-Hernandez T., Menéndez P. (2025). CAR-T Cell Therapy for Cancer: Current Challenges and Future Directions. Signal Transduct. Target. Ther..

[11] Haubner S., Subklewe M., Sadelain M. (2025). Honing CAR T Cells to Tackle Acute Myeloid Leukemia. Blood.

[12] Lloret-Madrid P., Chorão P., Guerreiro M., Montesinos P. (2025). CAR-T Cell Therapy for Acute Myeloid Leukemia: Where Do We Stand Now?. Curr. Oncol..

[13] Atilla E. (2026). Modulating CAR-T Cell Exhaustion and Fitness in Acute Myeloid Leukemia: Mechanistic Metabolic and Microenvironmental Strategies. Front. Immunol..

[14] Li Y.-R., Chen Y., Yang L. (2026). Exploring CAR Cell Therapies beyond CAR-T for Myeloid Malignancies. J. Biomed. Sci..

[15] Pérez-Amill L., Bataller À., Delgado J., Esteve J., Juan M., Klein-González N. (2023). Advancing CART Therapy for Acute Myeloid Leukemia: Recent Breakthroughs and Strategies for Future Development. Front. Immunol..

[16] Bras A.E., de Haas V., van Stigt A., Jongen-Lavrencic M., Beverloo H.B., te Marvelde J.G., Zwaan C.M., van Dongen J.J.M., Leusen J.H.W., van der Velden V.H.J. (2019). CD123 Expression Levels in 846 Acute Leukemia Patients Based on Standardized Immunophenotyping. Cytom. B Clin. Cytom..

[17] Van Oers F., Flumens D., Haentjens S., Krekelbergh L., Anguille S. (2026). The Search for Safe and Effective CAR-T Targets in AML. Blood Cancer J..

[18] Khalifeh M., Hopewell E., Salman H. (2025). CAR-T Cell Therapy for Treatment of Acute Myeloid Leukemia, Advances and Outcomes. Mol. Ther..

[19] Wang Y., Yu T., Wang M., Yu L. (2026). Immunological Barriers and Engineering Strategies for CAR-T Cell Therapy in Acute Myeloid Leukemia. Front. Immunol..

[20] Vanhooren J., Dobbelaere R., Derpoorter C., Deneweth L., Van Camp L., Uyttebroeck A., De Moerloose B., Lammens T. (2023). CAR-T in the Treatment of Acute Myeloid Leukemia: Barriers and How to Overcome Them. HemaSphere.

[21] van Gils N., Denkers F., Smit L. (2021). Escape From Treatment; the Different Faces of Leukemic Stem Cells and Therapy Resistance in Acute Myeloid Leukemia. Front. Oncol..

[22] Cuenca M., Sintes J., Lányi Á., Engel P. (2019). CD84 Cell Surface Signaling Molecule: An Emerging Biomarker and Target for Cancer and Autoimmune Disorders. Clin. Immunol..

[23] Pérez-Amill L., Armand-Ugón M., Val-Casals M., Martín-Herreros B., Álamo J.R., Peña S., Frigola G., Altuna A., Santos C., Guijarro F. (2025). A Novel Chimeric Antigen Receptor T-Cell Therapy Targeting CD84 for the Treatment of Acute Myeloid and T-Cell Lymphoblastic Leukemias. Leukemia.

[24] Pigazzi M., Marini O., Porcù E., Faggin G., Varotto E., Rizzardi P., Buldini B., Locatelli F., Biffi A. (2022). Preclinical Development of a CAR-T Cell Approach Targeting the CD84 Antigen Associated to Pediatric Acute Myeloid Leukemia. Blood.

[25] Pigazzi M., Merlini S., Da Ros A., Marini O., Faggin G., Fortuna N., Mattera R., Buldini B., Rizzardi P., Meshinchi S. (2026). CD84 Is a Specific Target for Acute Myeloid Leukemia CAR-T Cell Therapy. Nat. Commun..

[26] Zhu Y., Murtadha M., Liu M., Caserta E., Napolitano O., Nguyen L.X.T., Wang H., Moloudizargari M., Nigam L., Tandoh T. (2025). Identification of CD84 as a Potent Survival Factor in Acute Myeloid Leukemia. J. Clin. Investig..

[27] Kalina T., Flores-Montero J., van der Velden V.H.J., Martin-Ayuso M., Böttcher S., Ritgen M., Almeida J., Lhermitte L., Asnafi V., Mendonça A. (2012). EuroFlow Standardization of Flow Cytometer Instrument Settings and Immunophenotyping Protocols. Leukemia.

[28] van Dongen J.J.M., Lhermitte L., Böttcher S., Almeida J., van der Velden V.H.J., Flores-Montero J., Rawstron A., Asnafi V., Lécrevisse Q., Lucio P. (2012). EuroFlow Antibody Panels for Standardized N-Dimensional Flow Cytometric Immunophenotyping of Normal, Reactive and Malignant Leukocytes. Leukemia.

[29] Oporto Espuelas M., Kanannejad S., Michelozzi I.M., Gomez Castaneda E., Xenakis T., Kirtsios E., Macpherson H., Bartram J., Thomas R., Giacopuzzi E. (2024). Single Cell Multimodal Framework Identifies CD84 As a Leukaemic Stem Cell Target in Paediatric AML. Blood.

[30] Martín-Herreros B., Cordón L., Sargas C., Romero S., Linares M.D., Lloret P., Navarro-Vicente I., Cano I., Acuña-Cruz E., Torres-Miñana L. (2026). Prognostic Impact of Immunophenotypic Classification for Newly Diagnosed *NPM1*-mutated Acute Myeloid Leukemia. Cytom. B Clin. Cytom..

[31] Matarraz S., Leoz P., Yeguas-Bermejo A., van der Velden V., Bras A.E., Sánchez Gallego J.I., Lecrevisse Q., Ayala-Bueno R., Teodosio C., Criado I. (2023). Baseline Immunophenotypic Profile of Bone Marrow Leukemia Cells in Acute Myeloid Leukemia with Nucleophosmin-1 Gene Mutation: A EuroFlow Study. Blood Cancer J..

[32] Ung M., Etchin J., Halfond A., DiFazio J., Keschner Y., Pyclik A., Campbell A., Wang R., Silva M., Gjeci B. (2026). A Multimodal Atlas for Immunotherapeutic Targeting of AML Surface Heterogeneity. iScience.

[33] Azevedo R.S., Wang S.A., Swanson D.M., Daver N.G., Pemmaraju N., Kadia T.M., Issa G.C., Yilmaz M., Borthakur G., Senapati J. (2025). CD123 Expression and Its Correlation with Mutation Profile in Acute Myeloid Leukaemia. Br. J. Haematol..

