# Supplementary material for: CD84 Expression Across Disease Stages and Leukemic Subpopulations in Acute Myeloid Leukemia
Source: Cancers (Basel). 2026 Sep 18;18(18):3037. doi: 10.3390/cancers18183037 (PMC13604704; doi:10.3390/cancers18183037)
Supplement: Supplementary file 1 [file cancers-18-03037-s001.zip › cancers-4504659-supplementary.pdf]

Table S1. Monoclonal antibody information.

Antibody	Fluorochrome	Clone	Catalog number	Manufacturer
CD10	APC-H7	HB7	655404	Beckton Dickinson
CD11b	APC	D12	333143	Beckton Dickinson
CD13	PE-Cy7	L138	338439	Beckton Dickinson
CD14	APC-H7	MPϕ9	641394	Beckton Dickinson
CD15	FITC	MMA	332778	Beckton Dickinson
CD19	FITC	4G7	345788	Beckton Dickinson
CD33	PE-Cy7	P67.6	333952	Beckton Dickinson
CD34	PerCP-Cy5.5	8G12	347222	Beckton Dickinson
CD38	APC-H7	HB7	656646	Beckton Dickinson
CD45	V500-C	2D1	655873	Beckton Dickinson
CD64	FITC	10.1	555527	Beckton Dickinson
CD117	PE-Cy7	104D2D1	B49221	Beckman Coulter
CD123	APC	AC145	130-113-322	Miltenyi Biotec
CD300e	APC	UP-H2	656158	Beckton Dickinson
HLA-DR	V450	L243	655874	Beckton Dickinson

Table S2. Patient characteristics and CD84 (clone CD84.1.21) expression in the leukemic populations. Rows highlighted in grey indicate the same patient.

ID	Sample*	Timepoint	Age	Gender	Clone	Leukemic cells (%)	WHO 2022 classification	CD84 expression levels with clone CD84.1.21					
								Main population		Second population		Thrid population	
								%	nMedFI	%	nMedFI	%	nMedFI
2	BM	Refractory	45	Male	CD84.1.21	13	NR	74	22.4	100	57.8	NA	NA
3	BM	Diagnosis	77	Male	CD84.1.21	34	AML-MR	83	21.6	NA	NA	NA	NA
4	PB	Diagnosis	75	Female	CD84.1.21	3	AML-*NPM1*mut	100	17.6	NA	NA	NA	NA
5	BM/PB	Diagnosis	60	Male	CD84.1.21	65	AML-MR	100	100	98	32	2	3.5
7	BM/PB	Diagnosis	57	Female	CD84.1.21	59	AML with maturation	99	35	NA	NA	NA	NA
8	PB	Diagnosis	71	Male	CD84.1.21	42	AML-MR	69	22.2	NA	NA	NA	NA
8	BM	Refractory	71	Male	CD84.1.21 and 153-4D9	10		70	4.8	NA	NA	NA	NA
9	BM	Relapse	62	Male	CD84.1.21	14	NR	89	14.4	NA	NA	NA	NA
10	BM/PB	Diagnosis	63	Male	CD84.1.21	25	AML-MR (ICC: AML-TP53)	88	39.8	98	20.5	NA	NA
11	BM/PB	Diagnosis	53	Male	CD84.1.21	87	AML-*NPM1*mut	98	23.5	99	23.9	NA	NA
12	BM/PB	Diagnosis	72	Male	CD84.1.21	74	AML-MR	98	115.9	100	222.4	NA	NA
13	PB	Diagnosis	81	Male	CD84.1.21	15	AML-*NPM1*mut	99	20.6	NA	NA	NA	NA
14	BM/PB	Diagnosis	74	Male	CD84.1.21	81	AML-MR (ICC: AML-TP53)	93	38.4	99	67.4	37	6.3
20	BM/PB	Diagnosis	73	Male	CD84.1.21	78	AML-MR	93	11	NA	NA	NA	NA
21	PB	Refractory	44	Male	CD84.1.21	89	NR	88	15	NA	NA	NA	NA
22	BM/PB	Diagnosis	80	Male	CD84.1.21	58	AML-MR	66	43.2	NA	NA	NA	NA
23	BM/PB	Diagnosis	75	Female	CD84.1.21	43	AML-MR (ICC: AML-TP53)	34	3.8	NA	NA	NA	NA
24	BM	Diagnosis	79	Male	CD84.1.21	30	AML-MR (ICC: AML-TP53)	95	51.7	99	116.6	NA	NA
26	PB	Refractory	53	Female	CD84.1.21 and 153-4D9	29	NR	100	NV	NA	NA	NA	NA
27	BM	Relapse	48	Female	CD84.1.21	56	NR	96	16.4	NA	NA	NA	NA
28	PB	Relapse	49	Female	CD84.1.21 and 153-4D9	95	NR	97	NV	100	NV	NA	NA
29	BM/PB	Diagnosis	82	Male	CD84.1.21	28	AML with NUP98::PRRX2 rearrangement, post cytotoxic therapy	98	37.5	99	65.7	NA	NA
30	BM	Refractory	73	Female	CD84.1.21	91	AML MLLT10::KMT2A rearrangement	94	39.6	NA	NA	NA	NA
31	BM/PB	Diagnosis	67	Female	CD84.1.21	56	AML-*NPM1*mut	99	49.1	47	9.8	NA	NA
32	BM	Relapse	54	Male	CD84.1.21	85	NR	37	3.99	98	31.9	NA	NA
32	BM	Refractory	54	Male	CD84.1.21 and 153-4D9	40		97	33.8	0	1.6	NA	NA
33	BM/PB	Diagnosis	72	Female	CD84.1.21	34	AML-MR (ICC: AML-TP53)	95	46.5	NA	NA	NA	NA
33	BM	Refractory	72	Female	CD84.1.21 and 153-4D9	36		99	61.8	NA	NA	NA	NA
34	BM/PB	Diagnosis	74	Female	CD84.1.21	NR	AML-*NPM1*mut	99	44	98	23.3	NA	NA
35	BM	Refractory	44	Male	CD84.1.21	45	NR	99	62.6	97	53.9	NA	NA
36	BM/PB	Relapse	67	Female	CD84.1.21	37	NR	34	4.2	NA	NA	NA	NA

37	PB	Diagnosis	81	Male	CD84.1.21	57	AML-*NPM1*mut	100	28	NA	NA	NA	NA
38	BM/PB	Diagnosis	73	Male	CD84.1.21	24	AML-MR	100	57.7	99	2.9	NA	NA
39	BM	Refractory	45	Male	CD84.1.21	6	NR	100	38.8	77	28	NA	NA
40	BM	Relapse	77	Male	CD84.1.21	10	NR	85	12.1	NA	NA	NA	NA
41	BM	Relapse	38	Female	CD84.1.21	42	NR	99	19.5	NA	NA	NA	NA
42	BM/PB	Diagnosis	65	Male	CD84.1.21	90	NR	97	34.9	99	38.6	NA	NA
42	BM	Refractory	65	Male	CD84.1.21 and 153-4D9	38		96	21.3	92	19.8	NA	NA
43	BM	Refractory	70	Female	CD84.1.21	6	NR	96	35.8	NA	NA	NA	NA
44	BM	Refractory	76	Female	CD84.1.21	88	NR	98	55.7	NA	NA	NA	NA
45	PB	Diagnosis	80	Female	CD84.1.21 and 153-4D9	83	AML-*NPM1*mut	91	29.5	NA	NA	NA	NA
46	PB	Diagnosis	74	Female	CD84.1.21 and 153-4D9	16	NR	100	40.6	NA	NA	NA	NA
48	BM/PB	Diagnosis	64	Male	CD84.1.21 and 153-4D9	40	NR	96	3.9	42	1.9	NA	NA
49	PB	Diagnosis	72	Female	CD84.1.21 and 153-4D9	21	NR	100	40.3	NA	NA	NA	NA
50	BM/PB	Diagnosis	79	Female	CD84.1.21 and 153-4D9	55	AML-*NPM1*mut	99	30	NA	NA	NA	NA
52	BM	Diagnosis	61	Male	CD84.1.21 and 153-4D9	24	AML-MR (ICC: AML-TP53)	99	13.4	NA	NA	NA	NA
53	BM	Refractory	73	Male	CD84.1.21 and 153-4D9	60	NR	92	10.6	NA	NA	NA	NA
54	PB	Refractory	66	Male	CD84.1.21 and 153-4D9	38	NR	99	68	NA	NA	NA	NA
56	BM/PB	Diagnosis	32	Female	CD84.1.21 and 153-4D9	86	AML without maturation	100	68.5	NA	NA	NA	NA
58	PB	Diagnosis	57	Male	CD84.1.21 and 153-4D9	44	AML-MR	42	3,6	100	35.9	NA	NA
58	PB	Refractory	57	Male	CD84.1.21 and 153-4D9	68		90	25.3	15	1.5	NA	NA
60	BM	Refractory	53	Female	CD84.1.21 and 153-4D9	92	NR	79	49	NA	NA	NA	NA
61	BM/PB	Diagnosis	78	Male	CD84.1.21 and 153-4D9	42	AML-MR	99	48.7	NA	NA	NA	NA
62	PB	Relapse	76	Female	CD84.1.21 and 153-4D9	99	NR	100	38	NA	NA	NA	NA
65	BM	Diagnosis	60	Female	CD84.1.21 and 153-4D9	79	NR	100	160.8	NA	NA	NA	NA
69	BM/PB	Diagnosis	75	Male	CD84.1.21 and 153-4D9	40	AML-*NPM1*mut	91	23.2	100	16.21	NA	NA
70	BM/PB	Diagnosis	75	Male	CD84.1.21 and 153-4D9	71	AML-*NPM1*mut	100	41	NA	NA	NA	NA
71	BM/PB	Diagnosis	82	Female	CD84.1.21 and 153-4D9	33	AML with maturation	100	53.7	98	23.9	NA	NA
72	BM/PB	Diagnosis	59	Female	CD84.1.21 and 153-4D9	31	AML-*NPM1*mut	99	25.9	98	26.7	NA	NA
73	BM	Refractory	55	Male	CD84.1.21 and 153-4D9	94	NR	99	22.2	NA	NA	NA	NA
75	BM	Relapse	37	Female	CD84.1.21 and 153-4D9	25	NR	94	29.3	NA	NA	NA	NA

76	BM	Refractory	47	Female	CD84.1.21 and 153-4D9	69	NR	98	28.9	100	235.5	NA	NA
77	BM/PB	Diagnosis	68	Male	CD84.1.21 and 153-4D9 BM/ CD84.1.21 PB	24	AML-MR	98	24	NA	NA	NA	NA
78	BM	Relapse	40	Male	CD84.1.21 and 153-4D9	92	AML-KMT2A-rearrangement	100	21	NA	NA	NA	NA
79	BM	Relapse	53	Female	CD84.1.21 and 153-4D9	85	AML-KMT2A-rearrangement	91	8.13	100	12.9	NA	NA
80	BM	Diagnosis	68	Male	CD84.1.21 and 153-4D9	27	AML-MR	95	57.37	NA	NA	NA	NA

BM: bone marrow; PB: peripheral blood; NR: not reported; AML: acute myeloid leukemia; nMedFI: normalized median fluorescence intensity; NA: not applicable. * Bone marrow data are shown for cases in which both bone marrow and peripheral blood samples were processed.

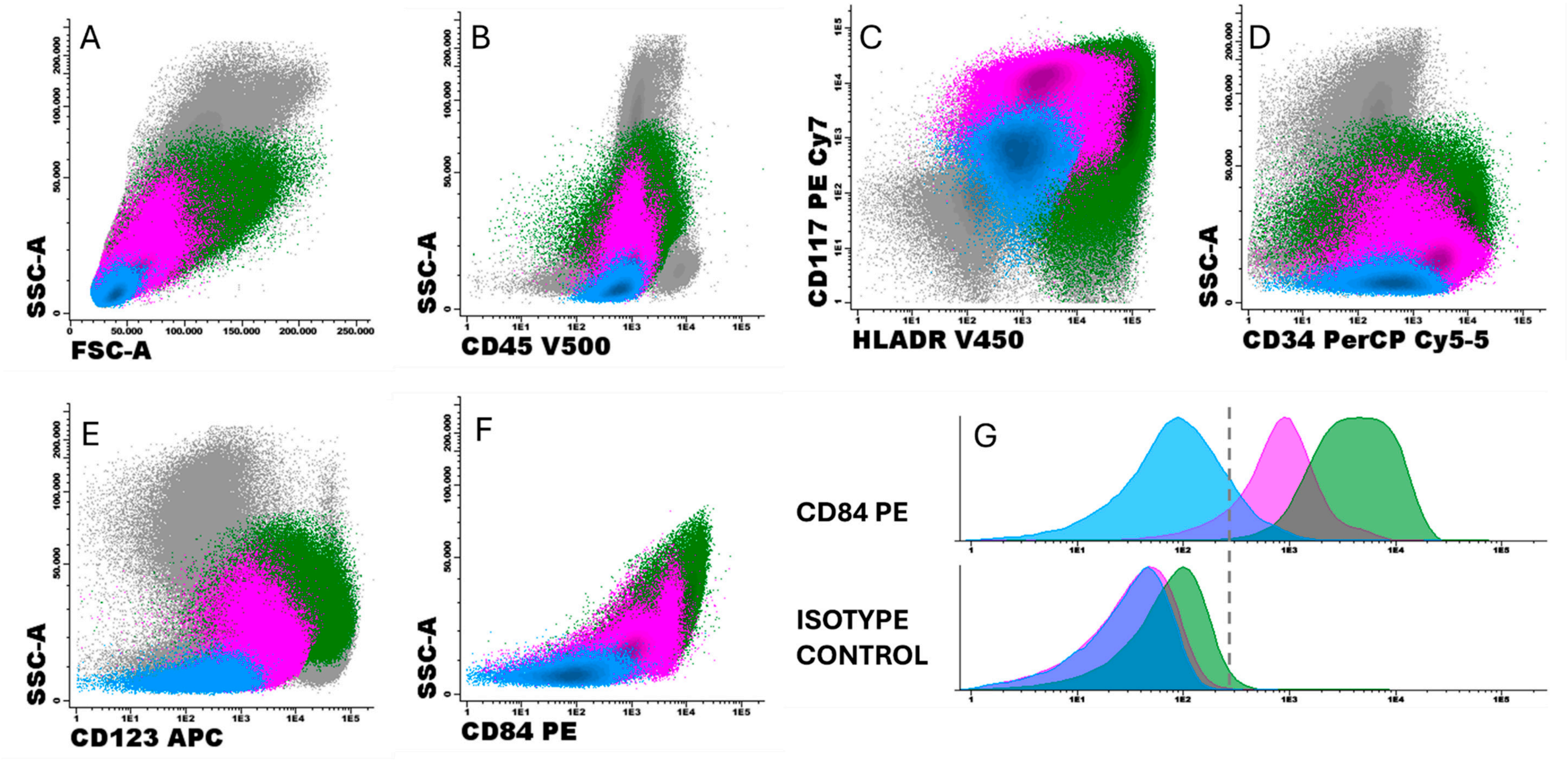

Figure S1. A representative flow cytometry image of the sequential gating strategy in a patient with three distinct leukemic populations: monocytic cells CD34$^{+ \text{ partial}}$ CD117 ^{heterogeneous} HLA-DR ^{high} (green), CD34^{+} CD117^{+} HLA-DR^{+} leukemic cells (magenta), and CD34$^{+ \text{ partial}}$ CD117$^{+ \text{ weak}}$ leukemic cells (blue). Dot plots show the distribution of leukocytes (grey) and leukemic populations according to FSC/SSC, CD45, HLA-DR, CD117, and CD34 expression (A-D). In this case, CD123 expression, among other markers, contributed to the identification and discrimination of the three leukemic

populations (E). The CD84 dot plot illustrates the differential expression of CD84 (clone CD84.1.21) across the leukemic populations (F). The histogram shows CD84 (clone CD84.1.21) expression relative to the isotype control for each population (G). The dashed line indicates the positivity cutoff.

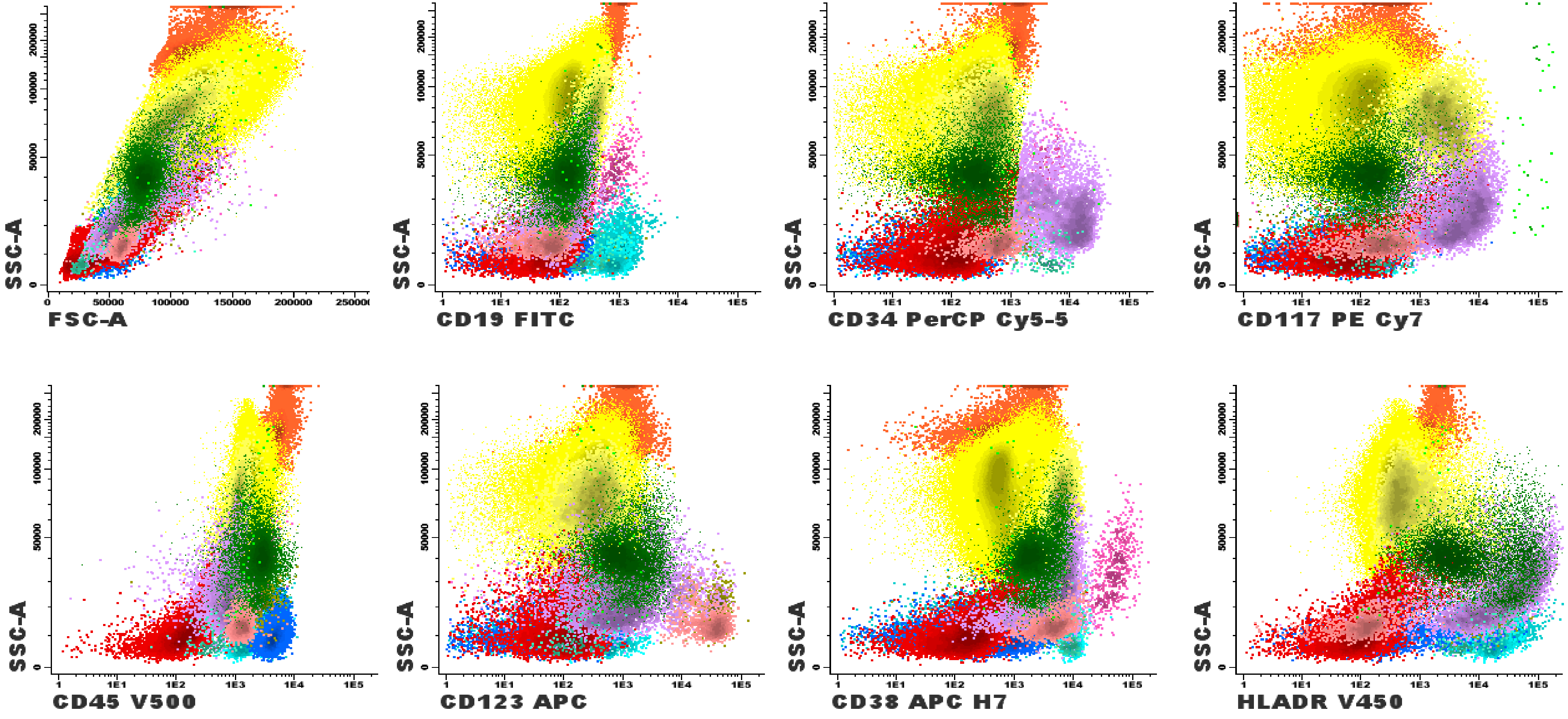

Figure S2. Representative flow cytometry plots showing the identification of the main normal hematopoietic populations in healthy bone marrow samples. Dot plots illustrate the distribution of leukocytes according to FSC/SSC characteristics and the expression of selected markers from the study panel used to discriminate the different hematopoietic populations. The identified populations were subsequently evaluated for CD84 expression. CD34+ myeloid progenitors (CD34^{+} CD117^{+}, lavender), monocytic cells (CD38^{+} HLA-DR $^{+heterogeneous}$ SSC-intermediate CD123^{+}, green), B lymphocytes (CD19^{+}, light blue), mast cells (CD117 $^{++\ high}$, light green), and basophils (CD123^{++} HLA-DR^{-}, pink) were identified were identified based on their characteristic immunophenotypic marker expression patterns as visualized throughout the panel dot plots.
